# Combining molecular patterns and clinical data for better immune checkpoint inhibitor prediction in metastatic urothelial carcinoma

**DOI:** 10.1007/s00262-025-04224-8

**Published:** 2025-11-12

**Authors:** M. Váradi, O. Horváth, E. Soós, A. Csizmarik, B. Németh, B. Győrffy, I. Kenessey, H. Reis, F. Koll, C. Oláh, B. Hadaschik, U. Krafft, V. Grünwald, F. Mairinger, M. Wessolly, M. J. Hoffmann, C. M. Grunewald, G. Niegisch, C. L. Cotarelo, A. Maráz, L. Kuthi, A. M. Szász, Z. Herold, M. Posta, B. Bátai, P. Nyirády, T. Szarvas

**Affiliations:** 1 Department of Urology, Semmelweis University, Budapest, Hungary; 2 Department of Genitourinary Medical Oncology and Pharmacology, National Institute of Oncology, Budapest, Hungary; 3 Department of Bioinformatics, Semmelweis University, Budapest, Hungary; 4 Department of Biophysics, University of Pecs, Pecs, Hungary; 5 HUN-REN Research Centre for Natural Sciences, Institute of Molecular Life Sciences, Budapest, Hungary; 6 Department of Pathology, Forensic and Insurance Medicine, Semmelweis University, Budapest, Hungary; 7 National Cancer Registry, National Institute of Oncology, Budapest, Hungary; 8 Dr. Senckenberg Institute of Pathology, Goethe University Frankfurt, Frankfurt Am Main, Germany; 9 Department of Urology, Goethe University, Frankfurt Am Main, Germany; 10 Department of Urology, University of Duisburg-Essen, Essen, Germany; 11 Clinic for Medical Oncology, Clinic for Urology, Interdisciplinary Genitourinary Oncology, University Hospital Essen, Essen, Germany; 12 Institute of Pathology, University of Duisburg-Essen, Essen, Germany; 13 Department of Urology, University Hospital and Medical Faculty of the Heinrich-Heine University, Düsseldorf, Germany; 14 Centre for Integrated Oncology (CIO) Düsseldorf, CIO Aachen-Bonn-Cologne-Düsseldorf, Düsseldorf, Germany; 15 Medical Faculty Mannheim, Institute of Pathology, Heidelberg University, Mannheim, Germany; 16 Institute of Pathology, University Medical Center of Heinrich-Heine University, Düsseldorf, Germany; 17 Department of Oncotherapy, University of Szeged, Szeged, Hungary; 18 Tumor Pathology Center, Department of Surgical and Molecular Pathology, National Institute of Oncology, Budapest, Hungary; 19 Department of Pathology and Experimental Cancer Research, Semmelweis University, Budapest, Hungary; 20 Department of Internal Medicine and Oncology, Division of Oncology, Semmelweis University, Budapest, Hungary; 21 Department of Pathology and Experimental Cancer Research, HCEMM-SU Molecular Oncohematology Research Group, Semmelweis University, Budapest, Hungary; 22 Department of Internal Medicine and Hematology, Semmelweis University, Budapest, Budapest, Hungary

**Keywords:** Urothelial carcinoma, Immune checkpoint inhibitors, Molecular subtypes, Gene expression based markers, PD-L1

## Abstract

**Background:**

The therapeutic landscape of advanced urothelial carcinoma (UC) is evolving, making the prediction of immune checkpoint inhibitor (ICI) therapy efficacy crucial. Standalone biomarkers offer limited predictive value, necessitating integrative approaches combining clinicopathological, laboratory, and molecular factors to enhance accuracy. This study aimed to evaluate clinical and molecular factors, including the real-life performance of PD-L1 IHC, to improve treatment outcome prediction in ICI-treated UC patients, ultimately developing a more precise therapy selection model.

**Methods:**

We conducted a retrospective, multicenter study on advanced or metastatic UC patients with available formalin-fixed, paraffin-embedded tumor samples who underwent ICI therapy (*n* = 100). NanoString technology was used to analyze 770 immune-related genes and a 60-gene panel for molecular subtype classification. Identified genes were validated in the IMvigor210 dataset. Whole tissue PD-L1 expression was assessed using the Dako 22C3 antibody.

**Results:**

Our findings show that PD-L1 IHC has limited predictive value for ICI response. However, among multigene molecular factors, the neuronal signature, MDA p53-like, and TCGA–luminal-infiltrated subtypes were linked to improved OS. Additionally, we identified and validated five novel ICI-predictive genes (*PSMB10, HLA-E, IRF7, CXCL12,* and *C3*), and by combining molecular and clinicopathological parameters, we developed a model with enhanced predictive value.

**Conclusions:**

Our real-life cohort analysis confirms the limitations of standalone biomarkers like PD-L1. We identified gene expression-based markers with strong prognostic and predictive value for ICI treatment outcomes.

**Supplementary Information:**

The online version contains supplementary material available at 10.1007/s00262-025-04224-8.

## Introduction

According to the estimates of GLOBOCAN, urothelial carcinoma (UC) accounts for over 600,000 new cases and 200,000 deaths annually [1]. Patients diagnosed with high-grade muscle-invasive UC experience high rates of disease recurrence, progression, and mortality despite undergoing multimodal treatment including radical cystectomy and/or neoadjuvant and adjuvant therapy [2, 3]. The 5-year overall survival (OS) rate in metastatic UC (mUC) is around 8% [4].

While platinum-based chemotherapy has been standard of care for many years, recent trials exploring immune checkpoint inhibitors (ICIs), the FGFR inhibitor erdafitinib, and antibody–drug conjugates (Enfortumab Vedotin (EV)) have substantially changed treatment algorithms [5]. ICI therapies—targeting programmed cell death 1 (PD-1) and PD-1 ligand (PD-L1)—raised high hopes as they have provided long-term survival in a subset of mUC patients. Despite their potential, ICIs elicit responses in only 20–30% of mUC patients, with durable responses (> 2 years) being even rarer [6]. The promising efficacy of ICIs has driven the development of new combination therapies, such as nivolumab with chemotherapy and pembrolizumab with EV, aiming to improve outcomes. This evolving treatment landscape enables a more individualized approach, emphasizing the need for biomarker-driven patient selection to optimize therapy choices. Despite these advances, ICI monotherapy remains a relevant option, highlighting the urgent need for better predictive markers beyond PD-L1 expression to identify patients most likely to benefit from ICIs.

While current guidelines recommend using PD-L1 immunohistochemical (IHC) testing before initiating ICI therapy in cisplatin-ineligible mUC patients [7], the predictive value of PD-L1 testing for treatment selection is limited. Clinical trials reported inconsistent results regarding the ICI predictive value of PD-L1 IHC [8–10], since many PD-L1-negative patients still respond to treatment, raising concerns about PD-L1 as a standalone biomarker [11].

In addition to PD-L1 expression, a series of other predictive biomarkers have been investigated. High tumor mutational burden (TMB) may enhance tumor immunogenicity and potentially enhances the tumor’s response to ICI therapy [12]. Accordingly, high TMB has been associated with improved ICI response and survival in mUC [8]. However, TMB remained a costly and controversial marker and has not been able to establish itself as a clinical predictive marker.

Additionally, gene expression-based molecular subtypes may also affect responses to ICIs. The consensus classification-based luminal and neuroendocrine-like tumors respond well, the TCGA classification highlighted the luminal-infiltrated subtypes [13, 14], while validation studies confirmed the luminal and neuronal subtypes as ICI sensitive [15, 16].

Furthermore, gene expression-based markers such as CD8 and immune signatures [17] showed limited results. The predictive value of standalone biomarkers seems to be restricted and can be influenced by various factors, including tumor microenvironment dynamics, treatment history, and interpatient variability. To address these challenges, the use of integrative approaches that combine various clinicopathological, molecular, and immune-related factors is essential to improve predictive accuracy.

Therefore, we conducted a multicenter data and sample collection, to assess the impact of a wide range of clinicopathological, laboratory and molecular (genetic) factors on treatment outcomes (survival, radiographic response) of ICI monotherapy in mUC. In addition, we examined how molecular subtypes relate to therapy responses and survival outcomes in patients undergoing ICI treatment. Our ultimate goal was to develop an integrated clinical and molecular model to enhance the precision of ICI therapy prediction.

## Material and methods

### Patient cohorts and data collection

**Fig. 1:**
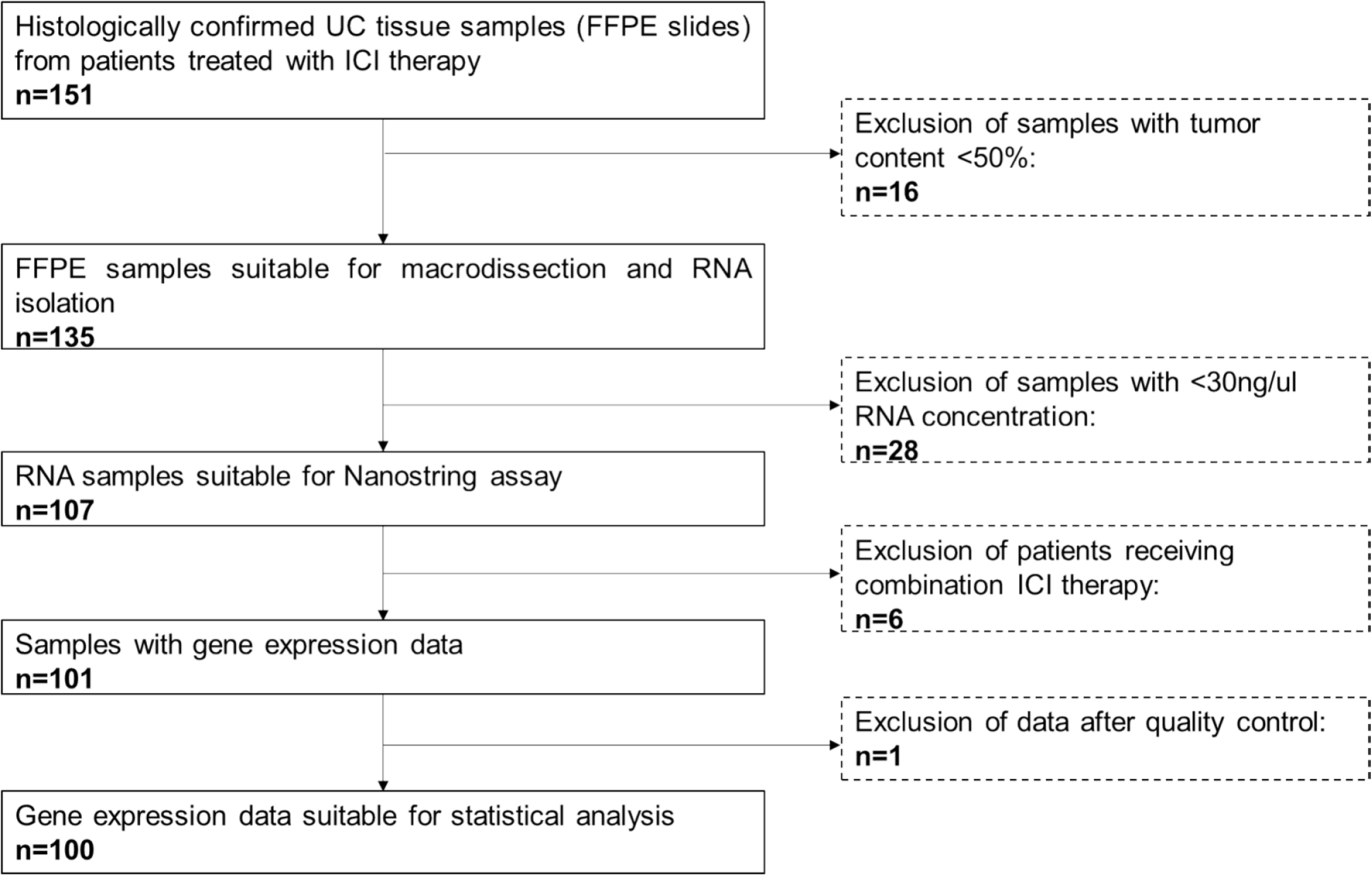
Flow-chart of the cohort selection

We conducted a retrospective, multicenter, real-world observational study of advanced or metastatic UC patients treated with immune checkpoint inhibitor (ICI) therapy (pembrolizumab or atezolizumab) as first- or second-line treatment between 01/2017 and 02/2023. Eligible patients had available FFPE primary tumor samples; those receiving combination ICI therapy, with poor-quality tissue, or insufficient follow-up were excluded (Fig. 1). Clinical, pathological, laboratory, and outcome data were extracted from medical records at six uro-oncology centers. According to the original and the enhanced Bellmunt risk score [18, 19], 10 g/dl, 30 mg/l, and 5 were used as the cut-offs for hemoglobin, C-reactive protein (CRP), and neutrophil–lymphocyte ratio (NLR), respectively. In addition, the lower limit of the normal range was employed as the cut-off value for albumin (35 g/l), and the upper limit of the normal range as the cut-off for LDH (250 U/l). For estimated glomerular filtration rate (eGFR), we applied the 40 ml/min as the cut-off value. The last update of follow-up was at 11/2023.

### RNA extraction and gene expression profiling

Tumor samples obtained via radical surgery (cystectomy or nephroureterectomy) or transurethral resection were processed for gene expression analysis. RNA was extracted from 10 μm FFPE tissue Sects. (2–10 per case) after macrodissection of tumor-rich areas (> 50% tumor content), as marked by a board-certified genitourinary pathologist on H&E-stained slides. RNA was isolated using the MagMAX™ FFPE DNA/RNA Ultra Kit (Thermo Fisher Scientific) per manufacturer’s instructions and quantified with the Qubit™ RNA HS Assay and Qubit fluorometer (Thermo Fisher Scientific).

RNA samples with sufficient yield (> 30 ng/μL) were hybridized for 24 h to the NanoString nCounter® Pan-Cancer IO 360™ panel and a custom panel (48 subtype-specific and 19 additional genes; STable 1). NanoString offers a robust platform that reliably analyzes even limited, degraded FFPE-derived RNA without extensive preprocessing. Samples were processed on the nCounter Prep Station, and expression was quantified using the nCounter Digital Analyzer and nSolver 4.0 software (NanoString).

### Molecular subtype classification

For molecular subtype classification according to the MDA, Lund, TCGA, and consensus classification systems, we used a gene panel-based approach with 48 genes covering six tumor cell-specific as well as three stroma-related gene signatures (STable 1) [20, 21].

### Immunohistochemistry

Whole tissue section PD-L1 expression was determined with Dako 22C3 antibody (Dako North America INC., Carpinteria, California, USA) according to manufacturer’s protocol. Tumor proportion scores (TPS), combined positive scores (CPS), and immune cell scores (IC) were assessed by one genitourinary pathologist blinded to all case characteristics. The cut-off values for PD-L1 positivity were as follows: TPS ≥ 5%, CPS ≥ 10, IC ≥ 5%.

### Statistical analysis

A detailed description of the statistical analysis is presented in the Supplementary Materials.

## Results

### Cohort and follow-up characteristics

Clinicopathological and follow-up data from 100 eligible mUC patients were analyzed (Table 1A). The median age at ICI-baseline was 70.3 years (range: 30.9–88.8 years). The original Bellmunt risk calculation was possible for 97% of the patients [18]. PD-L1 IHC could be evaluated in 77 cases. A Sankey diagram illustrating the clinical course of the 100 UC patients is provided in the Supplementary Materials (SFig. 1).

**Table 1:** Patients, treatment (A), and follow-up (B) characteristics for the whole cohort and by treatment lines

A				
Variables		Whole cohort	1L	2L
		*n* (%)	*n* (%)	*n* (%)
Total number of patients		100	30	70
Age at ICI initiation, years median [range]		70.3 [30.9–88.8]	72.1 [46.8–88.4]	68.8 [30.9–88.8]
Sex	Male	73 (73)	19 (63)	54 (77)
	Female	27 (27)	11 (37)	16 (23)
Location of primary tumor	UTUC	14 (14)	3 (10)	11 (16)
	BC	79 (79)	25 (83)	54 (77)
	Both	7 (7)	2 (7)	5 (7)
Setting	1L	30 (30)	30 (100)	0 (0)
	2L	70 (70)	0 (0)	70 (100)
Drug	Atezolizumab	34 (34)	8 (27)	26 (37)
	Pembrolizumab	66 (66)	22 (73)	44 (63)
Reason for 2L ICI	Progression	55 (55)	-	55 (79)
	Chemo intolerance	15 (15)	-	15 (21)
ECOG PS at ICI initiation	0	56 (56)	16 (53)	40 (57)
	1	33 (33)	12 (40)	21 (30)
	2	9 (9)	2 (7)	7 (10)
	NA	2 (2)	0 (0)	2 (3)
Metastatic sites	Only LN	29 (29)	10 (33)	19 (27)
	Liver	17 (17)	2 (7)	15 (21)
	Visceral	51 (51)	14 (47)	37 (53)
	Bone	24 (24)	2 (7)	22 (31)
Stage	T1	13 (13)	3 (10)	10 (14)
	T2	36 (36)	10 (33)	26 (37)
	T3	29 (29)	8 (27)	21 (30)
	T4	15 (15)	6 (20)	9 (13)
	NA	7 (7)	3 (10)	4 (6)
Histological variants	pure UC	75 (75)	24 (80)	51 (73)
	UC-SD	14 (14)	3 (10)	11 (16)
	UC-GD	1 (1)	1 (3)	0 (0)
	UC-MP	6 (6)	0 (0)	6 (9)
	UC-NE	1 (1)	1 (3)	0 (0)
	UC-LEL	1 (1)	0 (0)	1 (1)
	UC-SaV	2 (2)	1 (3)	1 (1)
Lymphovascular invasion	Yes	22 (22)	6 (20)	16 (23)
	No	12 (12)	4 (13)	8 (11)
	NA	66 (66)	20 (67)	46 (66)
Bellmunt risk factors (original)	0	40 (40)	11 (37)	29 (41)
	1	43 (43)	15 (50)	28 (40)
	2–3	14 (14)	3 (10)	11 (16)
	NA	3 (3)	1 (3)	2 (3)
PD-L1 IHC	CPS ≥ 10	46 (46)	14 (47)	32 (46)
	CPS < 10	31 (31)	8 (27)	23 (33)
	NA	23 (23)	8 (27)	15 (21)
	IC ≥ 5%	47 (47)	14 (47)	33 (47)
	IC < 5%	30 (30)	8 (27)	22 (31)
	NA	23 (23)	8 (27)	15 (21)
	TPS ≥ 5%	38 (38)	12 (40)	26 (37)
	TPS < 5%	39 (39)	10 (33)	29 (41)
	NA	23 (23)	8 (27)	15 (21)

B			
Variables	Whole cohort	1L	2L
	*n* (%)	*n* (%)	*n* (%)
Time of follow-up, months, median [range]	12.0 [0.5–81.9]	12.8 [0.5–81.9]	11.7 [0.5–58.3]
*Best overall response*			
Complete response	3 (3)	3 (10)	0
Partial response	31 (31)	6 (21)	25 (36)
Stable disease	24 (24)	7 (24)	17 (24)
Progressive disease	41 (41)	13 (45)	28 (40)
No radiologic evaluation performed	1	1	0
ORR	34 (34)	9 (31)	25 (36)
DCR	58 (58)	16 (55)	42 (60)
PFS, months, median [95% CI]	4.9 [3.4–8.6]	6.5 [2.9–13.8]	4.6 [3.1–8.6]
Death at last follow-up	75 (75)	22 (73)	53 (76)
OS, months, median [95% CI]	13.6 [9.1–18.4]	13.7 [8.7–30.9]	12.2 [7.6- 23.4]

ICI: immune checkpoint inhibitor, UTUC: upper tract urothelial carcinoma, BC: bladder cancer, 1L: first-line, 2L: second-line, NAC: neoadjuvant chemotherapy, ECOG-PS: Eastern Cooperative Oncology Group performance status, LN: lymph node, UC: urothelial carcinoma, UC-SD: UC with squamous differentiation, UC-GD: UC with glandular differentiation, UC-MP: Micropapillary variant, UC-SaV: Sarcomatoid variant, UC-NE: Neuroendocrine carcinoma, UC-LEL: Lymphoepithelioma, PD-L1: programmed death-ligand 1, IHC: immunohistochemistry, CPS: combined positive score, IC: immune cell, TPS: tumor proportion score, ORR: overall response rate, DCR: disease control rate, DOR: duration of response, PFS: progression-free survival, OS: overall survival, CI: confidence interval, NA: not available

The median follow-up period after ICI initiation was 12.0 months. Among the 99 patients evaluable for tumor response, three achieved complete remission (CR) and 31 showed partial response (PR), resulting in an objective response rate (ORR) of 34%. Disease control was achieved in 58 patients. The median PFS was 4.9 months. Seventy-five patients died during the follow-up, and the median OS was 13.6 months (Table 1B).

### Clinicopathological factors associated with survival and/or radiographic response

In the univariate Cox regression analyses, the following clinicopathological or laboratory factors were identified to be significantly correlated with poor OS: ECOG PS (> 1), the presence of liver or bone metastasis or any Bellmunt risk factor (1 +), low (< 10 g/dl) baseline hemoglobin levels, high (≥ 250 U/l) LDH levels, and impaired renal function (eGFR < 40 ml/min) (STable 2). To account for the potential confounding effect of therapy line, we included it as a covariate in our multivariate model; in this analysis, ICI setting was not independently associated with overall survival (HR 0.796, 95% CI 0.334–1.900, *p* = 0.608) (STable 3).

Considering PFS, the presence of liver metastasis, Bellmunt risk groups (1 +), low hemoglobin or albumin, and elevated LDH levels were associated with poor PFS (STable 2).

Therapy response data were available for 99 patients. Bone metastasis, Bellmunt risk factors and hemoglobin levels showed significant associations with both ORR and disease control rate (DCR). Additionally, age at ICI initiation, the presence of liver metastasis, and CRP values had significant correlation with DCR (STable 2). Stratified analyses of DCR and ORR by treatment line demonstrated broadly consistent associations across 1L and 2L patients, though some predictors differed in significance (STable 4).

### PD-L1 IHC

**Fig. 2:**
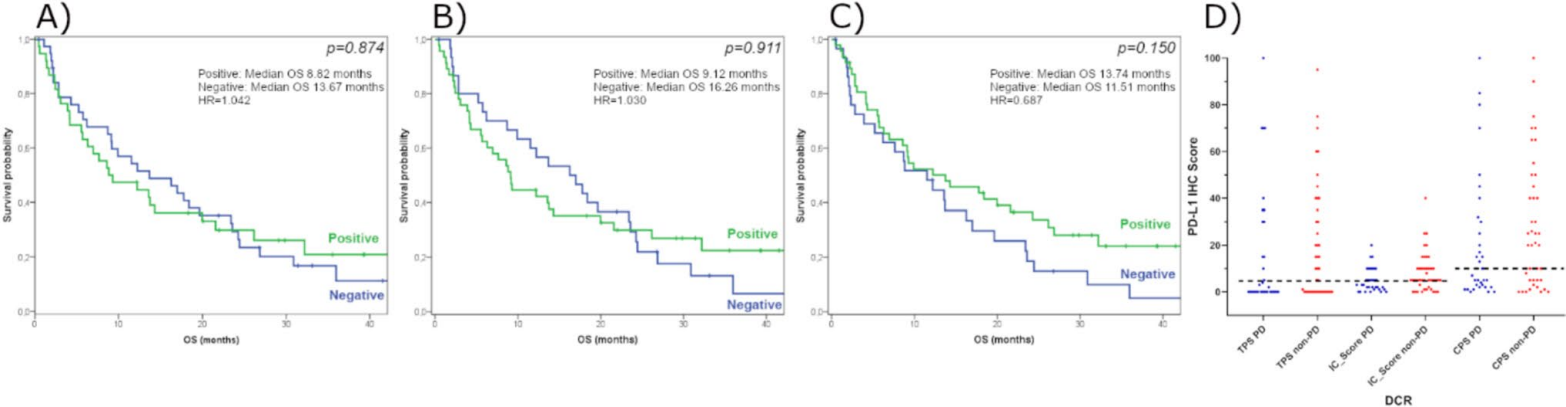
Kaplan–Meier survival plots for PD-L1 IHC positive vs. negative groups using **A** TPS, **B** CPS, **C** IC scoring systems and scatter plot for PD-L1 IHC scores in DCR groups (**D**)

The PD-L1 IHC analysis has been performed in a subcohort of 77 patients. No significant correlation was found between PD-L1 IHC positivity and survival (Fig. 2). Among the three different scoring systems evaluated, only the PD-L1 IC positivity showed a significant association with radiographic response (DCR but not ORR) (STable 5).

The specificity of the three scoring systems (CPS, TPS, IC) ranged between 50–60%, while their sensitivity values were around 60–70%. In tests used to guide treatment decisions, the negative predictive value (NPV) is a crucial measure; a higher NPV reduces the likelihood of withholding treatment from a patient who might benefit from it. The NPVs of PD-L1 IHC in our analyses were 54.8, 53.8, and 60.0%, respectively. Overall, among the applied PD-L1 evaluation methods, the IC scoring system proved to be the most promising, demonstrating the highest sensitivity, specificity, PPV, and NPV. However, the association of PD-L1 IC score with outcomes was limited, as it was significant only with DCR but not with OS or ORR, thereby restricting its value as a standalone decision-making marker. (Table 2).

**Table 2:** Specificity, sensitivity and predictive values of PD-L1 IHC test and CD274 gene expression test (optimal cut-off = 44) for the institutional and comparable RCT cohorts [9, 32]

PD-L1 IHC score		Institutional cohort		Comparable RCT			
		DCR	ORR	DCR	ORR	DCR	ORR
				*KEYNOTE-045*		*KEYNOTE-052*	
CPS	Specificity (%)	50.0	40.8	71.8	74.2	80.1	76.0
	Sensitivity (%)	67.4	60.7	24.0	28.1	43.5	50.0
	PPV (%)	63.0	37.0	40.3	25.8	71.2	50.0
	NPV (%)	54.8	64.5	54.3	76.3	55.8	76.0
TPS	Specificity (%)	61.8	53.1	–	–	–	–
	Sensitivity (%)	58.1	53.6	–	–	–	–
	PPV (%)	65.8	39.5	–	–	–	–
	NPV (%)	53.8	66.7	–	–	–	–

				*IMvigor210*		*IMvigor211*	
IC	Specificity (%)	52.9	42.9	72.3	72.5	74.7	75.0
	Sensitivity (%)	72.1	67.9	40.4	57.8	30.6	33.3
	PPV (%)	66.0	40.4	48.8	30.2	34.4	28.1
	NPV (%)	60.0	70.0	65.0	89.3	71.3	79.3
*CD274* GE	Specificity (%)	38.2	32.7	–	–	–	–
	Sensitivity (%)	74.4	71.4	–	–	–	–
	PPV (%)	60.4	37.7	–	–	–	–
	NPV (%)	54.2	66.7	–	–	–	–

CPS: combined positive score, TPS: tumor proportion score, IC: immune cell, GE: gene expression, PPV: positive predictive value, NPV: negative predictive value, DCR: disease control rate, ORR: objective response rate

**Fig. 3:**
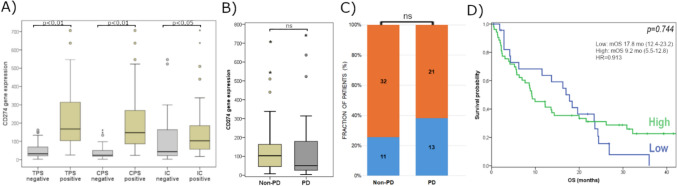
mRNA expression of CD274 in PD-L1 negative vs. PD-L1 positive subgroups (**A**) and in Non-PD vs. PD groups (**B**), CD274 gene expression (high—orange; low—blue) in non-PD and PD subgroups (**C**), Kaplan–Meier survival plots for CD274 gene expression groups (**D**)

Comparing PD-L1 protein and mRNA (*CD274* gene expression) levels, we found a strong correlation between *CD274* mRNA levels and PD-L1 IHC results when using the TPS or CPS scores (Pearson’s correlation coefficient: TPS, *r* = 0.642, *p* < 0.01; CPS, *r* = 0.597, *p* < 0.01) but not when considering the IC score (*p* = 0.959). Additionally, a significant association was found between PD-L1 IHC positivity and *CD274* gene expression levels across all three scoring systems (*p* < 0.01 for TPS and CPS, *p* = 0.049 for IC) (Fig. 3A).

Moreover, *CD274* gene expression did neither show a significant association with radiographic response (*p* = 0.589 for ORR and *p* = 0.259 for DCR) nor survival (*p* = 0.744) (Fig. 3B–D). Although testing *CD274* gene expression exhibited slightly lower sensitivity values, the specificity and predictive values were similar when compared to those of the PD-L1 IHC test (Table 2).

When comparing the gene expression profiles between PD-L1 IHC-positive *vs.* negative cases, we identified 245, 202, and 294 genes with significantly different expression levels for the TPS, IC, and CPS scores, respectively (STable 6).

### Correlation of molecular subtypes with survival and radiographic response

**Fig. 4:**
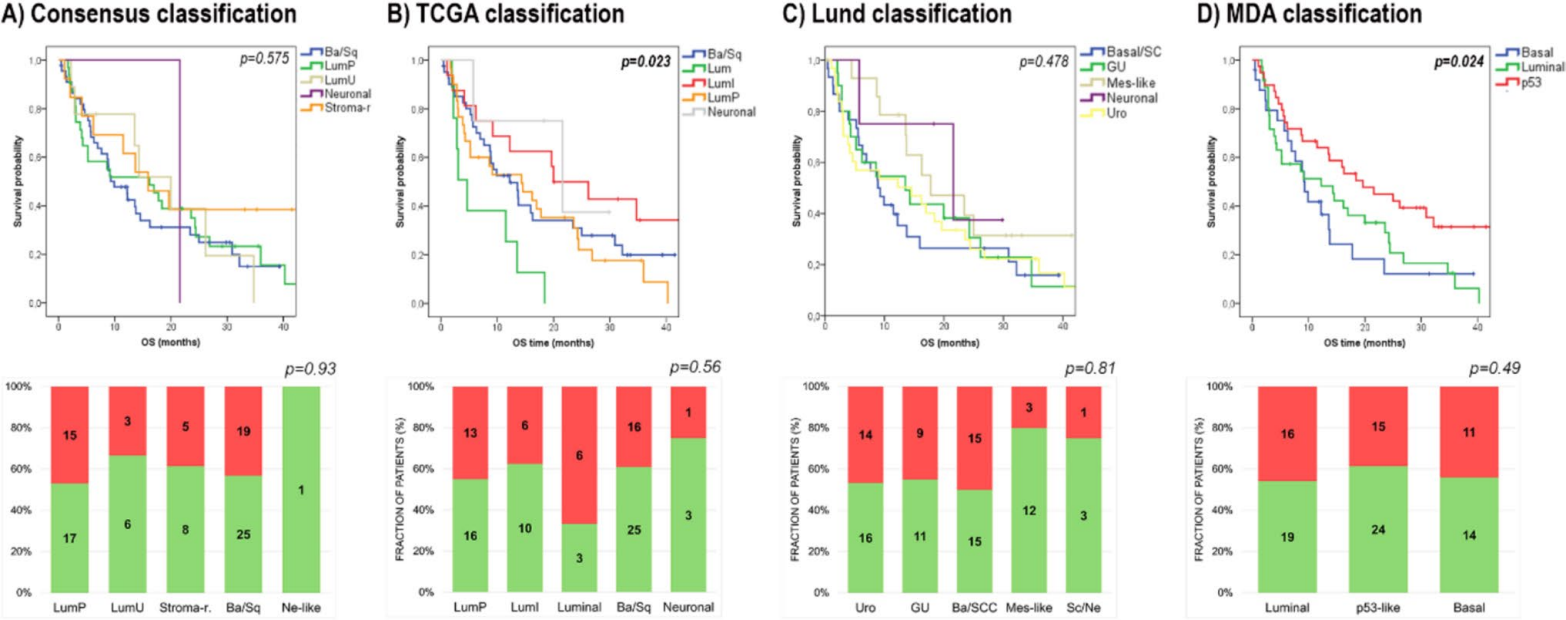
Survival and radiographic response (green: non-PD, red: PD) of different molecular subtypes according to the **A** consensus, **B** TCGA, **C** Lund, and **D** MDA classification systems

**Fig. 5:**
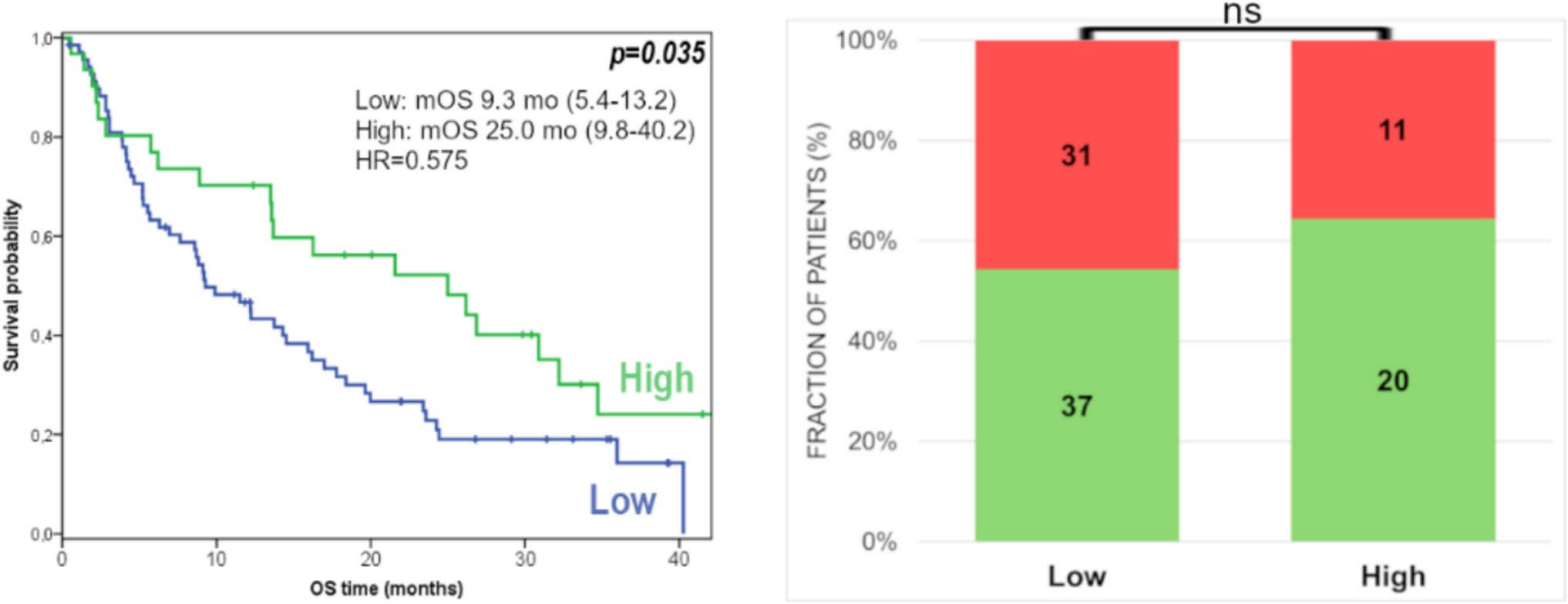
Survival and radiographic response (green: non-PD, red: PD) of patients with low (< 3.2) versus high (> 3.2) neuronal signature score

We identified molecular subtypes based on the MDA, TCGA, Lund, and consensus classification systems. No significant differences could be observed between the OS and PFS of the consensus and Lund-classification subgroups (*p* = 0.575 (OS) and *p* = 0.441 (PFS) for consensus, and *p* = 0.575 (OS) and *p* = 0.441 (PFS) for Lund, respectively). Nevertheless, patients in the MDA p53-like subgroup exhibited significantly longer OS (*p* = 0.024) and PFS (*p* = 0.043) compared to those in the luminal and basal subgroups. According the TCGA classification system, OS (*p* = 0.023) and PFS (*p* = 0.038) of the luminal subgroup proved to be inferior to the other groups. In addition, the neuronal and luminal-infiltrated subtypes exhibited longer OS; however, the case numbers in the neuronal group were low (Fig. 4). Among the evaluated signature scores, high neuronal scores (≥ 3.2), calculated from the expression levels of 10 genes—*APLP1, CHGB, ENO2, GNG4, MSI1, PEG10, PLEKHG4B, RND2, SV2A*, and *TUBB2B*—were associated with improved OS and PFS (Fig. 5).

In response rates of different molecular subtypes mainly due to the low case numbers of some subgroups no significant associations could be determined; however, some minor differences were observed. The TCGA–luminal subtype displayed the lowest response rate (Fig. 4). Furthermore, the proportion of responders was higher in patients with high neuronal signature scores (Fig. 5).

### Differential gene expression analysis

#### Survival and differentially expressed genes

The overall 95 genes associated with OS are shown in the heatmap in SFig. 3. Twenty-three of the 95 genes remained significant after FDR correction (Table 3). The subsequent validation with online tool KM Plotter in the transcriptome dataset of the IMvigor210 study (second-line atezolizumab in > 300 mUC patients) confirmed the prognostic value of 6 genes (*CR2, NCR1, HLA-E, IRF7, PSMB10* and *PSMB8*) (Table 3).

**Table 3:** List of genes with significant prognostic value and their immune response category according to NanoString annotation

Gene name	FDR	HR	95% CI	Cut-off value	Immune response category (NanoString® annotation)
*C1QBP*	0.019	2.44	1.35–4.40	1118	Chemokines
*MAP2K4*^*#*^	0.003	2.37	1.47–3.83	268	Innate immune response
*IL4*	0.030	0.71	0.45–1.12	4	Interleukins, regulation, T-cell functions
CR2*	0.035	0.64	0.40–1.02	18	Innate immune response, B-cell functions
*MBL2*^*#*^	0.028	0.57	0.35–0.92	10	Innate immune response
*IL12A*^*#*^	0.043	0.56	0.34–0.87	8	Adaptive immune response, cytokines, interleukins, NK cell functions, regulation, T-cell functions
*ROPN1*	0.019	0.55	0.33–0.93	10	–
NCR1*^#^	0.021	0.55	0.32–0.74	12	Cell Functions, NK Cell Functions
*DMBT1*	0.037	0.55	0.32–0.88	7	Innate immune response
*ELANE*	0.019	0.54	0.32–0.83	2	Regulation
*ENTPD1*	0.046	0.53	0.31–0.85	257	Adaptive immune response
*RB1*	0.020	0.52	0.32–0.86	610	–
*CHUK*	0.025	0.51	0.28–0.78	388	Innate immune response
HLA-E*^#^	0.047	0.51	0.30–0.85	3343	Regulation
IRF7*^#^	0.016	0.49	0.30–0.78	324	Innate immune response
PSMB10*^#^	0.041	0.48	0.29–0.79	727	Humoral immune response
*CD63*	0.021	0.47	0.28–0.78	15,453	–
PSMB8*^#^	0.029	0.47	0.28–0.78	850	Chemokines
*CXCL12*^*#*^	0.012	0.45	0.27–0.76	656	Chemokines
*IL4R*	0.004	0.43	0.25–0.72	570	Cytokines, T-cell functions
*ICAM2*	0.016	0.42	0.26–0.69	126	Adhesion, regulation
*MFGE8*	0.018	0.41	0.23–0.73	2125	Transporter functions
*C3*	0.007	0.39	0.23–0.67	1624	Innate immune response, regulation

*validated in IMvigor210 transcriptome dataset, #validated in KMPlotter pan-cancer dataset

#### Radiographic response and differentially expressed genes

**Fig. 6:**
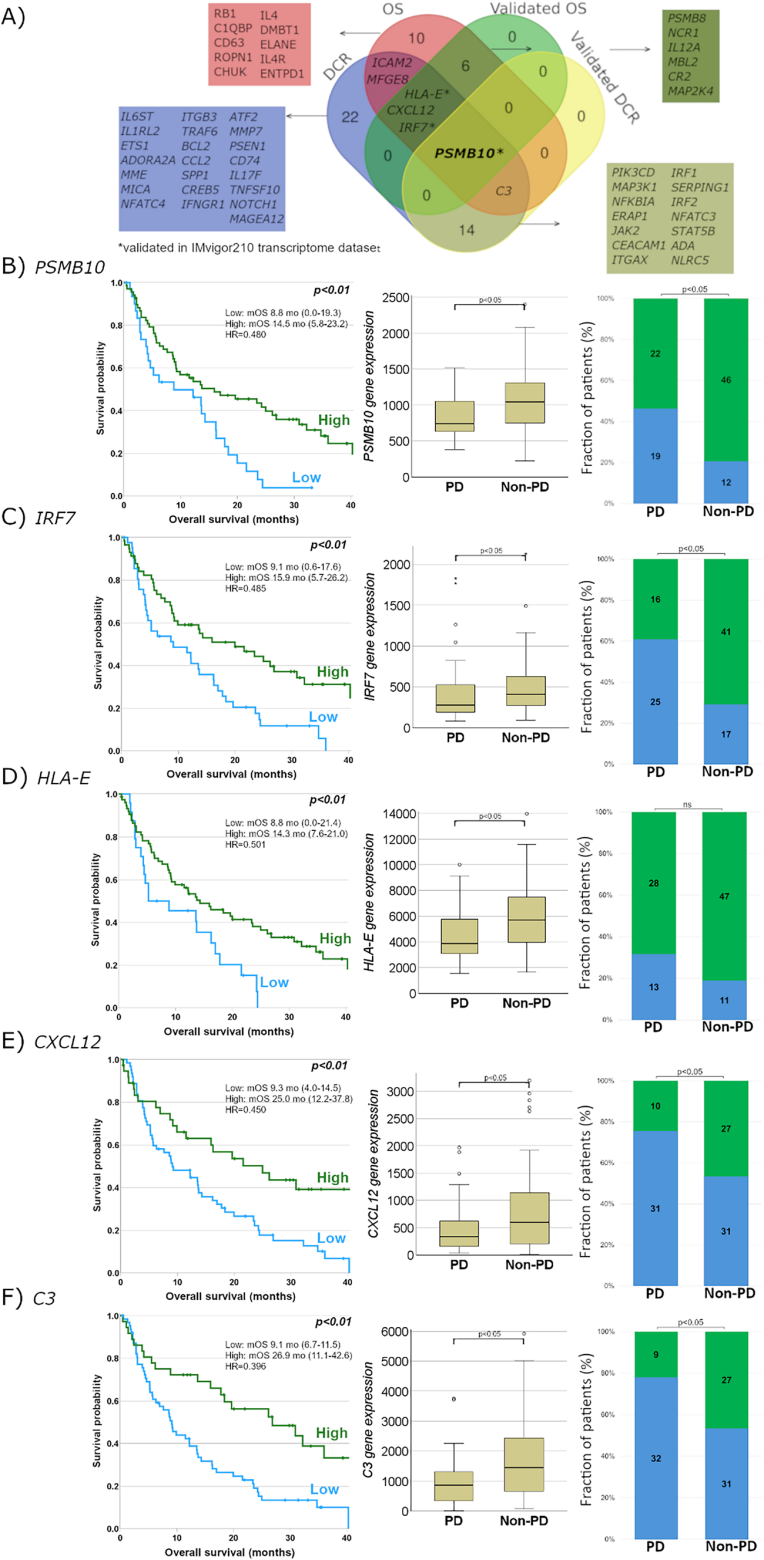
Clinical outcomes based on gene expression markers. **A** Venn diagram showing overlapping associations of genes with different endpoints. Response and OS based on **B** PSMB10 **C** IRF7, **D** HLA-E, **E** CXCL12, **F** C3 gene expression. Blue bar: low gene expression, green bar: high gene expression

Wilcoxon test identified 20 genes (as continuous variable) to be significantly associated with good or poor ORR (good: *MAGEA12, SPP1, GPI, ENO2, TFRC, BST1, C3, MME, MAGEA3, PRAME, NFKBIA,* and *CTAG1B*; poor: *TMEFF2, FLT4, ABCB1, CCL24, CREBBP, CD209, C8G,* and *MYD88*), and 43 genes to be associated with DCR (SFig. 3). ROC Plotter validation in the pan-cancer dataset identified 16 DEGs (STable 7). Of these, two genes, *IRF1* and *PSMB10,* also had significantly different expression levels in the UC transcriptome dataset (IMvigor210). The *PSMB10* gene was the only gene validated for both endpoints: DCR and OS. (Fig. 6).

#### Clinicopathological/laboratory parameters and differentially expressed genes

We found an overall number of 476 DEGs in various dichotomized clinicopathological or laboratory parameter groups. Notably, the highest number of DEGs (*n* = 202) was identified between PD-L1 IC positive ( +) versus negative (–) groups followed by bone metastasis (*n* = 124) and baseline hemoglobin levels (*n* = 95). Almost all of the IMvigor210 validated prognostic genes (PSMB10, PSMB8, IRF1, IRF7, HLA-E, and NCR1) showed significantly different GE between PD-L1 IC groups. Interestingly, the gene TGFB2 displayed significantly lower expression in advanced age group (68 < years), that may explain significantly better DCR in group of elderly patients. The list of DEGs is presented in STable 8. We also provided the RNA expression matrix (> 700 genes) together with the corresponding clinical data for the patients included in this study as STable 9.

#### Molecular subgroups and differentially expressed genes

The list of differently expressed genes between molecular subgroups is presented in STable 10. The highest number of DEGs (*n* = 406) was identified between MDA luminal versus MDA-p53 subgroups followed by TCGA-Ba/Sq versus LumP (*n* = 373), Lund–Basal versus GU (*n* = 263), and Consensus–Basal versus LumP (*n* = 238). Notably, no comparisons yielded differences in GE levels for the *PSMB10* gene. Meanwhile, all the other aforementioned IMvigor210 validated genes exhibited differential expression across some molecular subgroups.

### Prognostic model combining clinical and genomic factors for OS

**Fig. 7:**
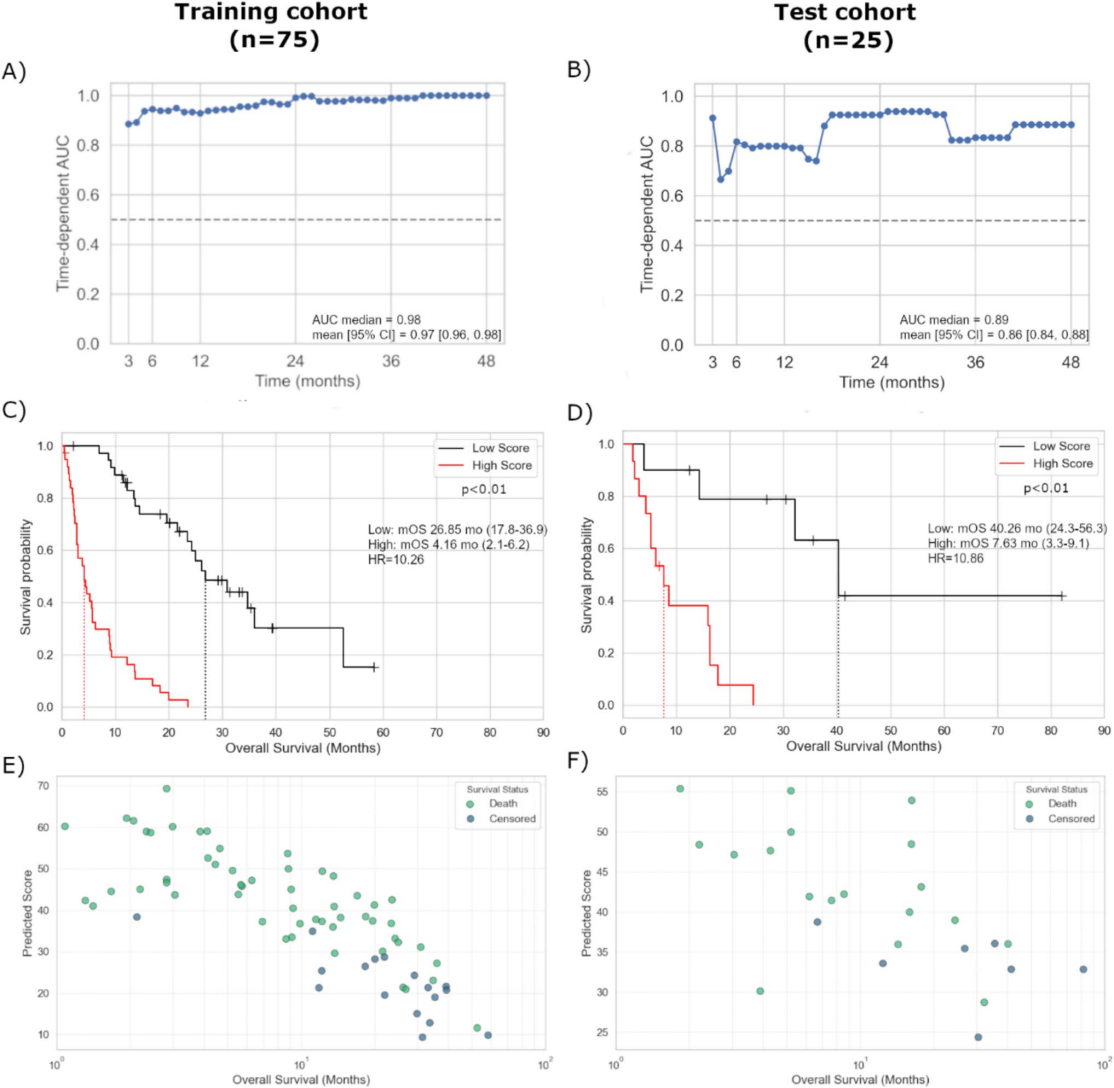
The best combination model’s performance and survival analysis: ROC Curves (**A**, **B**), Kaplan–Meier plots (**C**, **D**), and Survival vs. score scatter plot (**E**, **F**)

We utilized the Random Survival Forest model to identify the most effective prognostic model by combining clinicopathological and molecular factors that were significantly associated with OS in univariate analysis. An additional inclusion criterion was that each parameter had to be available for at least 80% of the patients to ensure adequate sample size. The dataset was randomly split into a training cohort (75%) to develop the prediction algorithm and a test cohort (25%) to assess the performance of the trained classifier. Both the area under the receiver operating characteristic (ROC) curve (AUC) and the concordance index (c-index) were calculated to evaluate the predictions. Our final model incorporated four laboratory parameters (LDH, Hg, eGFR, NLR) and 9 gene expression markers (*CR2, HLA-E, IRF7, PSMB10, PSMB8, CXCL12, IL12A, MAP2K4, IRF1*) and a gene expression signature (neuronal signature score). The median AUC for the prediction model (test cohort) was 0.89 (Fig. 7B), while the c-index in the test set was 0.77, superior to those of the standalone components (STable 11). Each patient was assigned a score based on the prediction model, and using the median score (38.5), they were classified into low-score and high-score groups. The median OS of the high-score group was significantly worse than that of the low-score group (7.6 *vs.* 40.3 months, HR = 10.86, *p* = 0.002 for the test cohort) (Fig. 7D).

When testing the predictive performance of the model, there were significant correlations with DCR and ORR (*p* < 0.01). The model’s NPV for ORR reached 84%, which is superior to that of PD-L1 IHC.

## Discussion

In the present study, we evaluated various clinical and molecular parameters for their prognostic and predictive value, in a real-world, multicenter cohort of ICI-treated mUC patients. Our findings indicate that PD-L1 IHC has only a limited ICI-predictive value. However, among multigene molecular factors, the neuronal signature, MDA p53-like, and TCGA–luminal-infiltrated subtypes proved to be associated with better OS. Additionally, we identified and validated five novel single ICI-predictive genes (*PSMB10, HLA-E, IRF7, CXCL12, C3*) and developed a model with excellent ICI-predictive value by combining molecular and clinicopathological parameters.

The therapeutic landscape of advanced UC is constantly expanding; thus, prediction of ICI efficacy has become increasingly essential. As standalone biomarkers display a limited predictive value integrative approaches that combine clinicopathological, laboratory, and molecular factors are essential to enhance predictive accuracy.

Several previous clinical trials and real-life studies have demonstrated that liver and bone metastases are strong prognostic and predictive factors in UC treated with ICIs [22–24]. A recent meta-analysis found that urological tumors with liver metastases have the worst prognosis among various cancers [25]. The real-life cohort analysis of Makrakis et al*.* further demonstrated that bone and liver metastases are linked to lower ORR, shorter PFS, and OS in UC patients treated with ICIs [26]. Our findings align with and further validate these established prognostic factors. Similarly, low baseline hemoglobin levels proved to be significantly associated with worse outcomes (ORR, DCR, OS, PFS) in our cohort. These results also support previous findings in the literature [27, 28].

PD-L1 is an easily detectable target of ICI therapy. Previous studies reported PD-L1 expression to be present in 20–30% UCs, often linked to more advanced disease stages [29]. Most studies suggested high PD-L1 expression in tumor cells to be associated with worse prognosis, its expression in immune cells correlated with a favorable prognosis [30]. Additionally, the role of PD-L1 expression as an ICI predictive biomarker remains controversial due to highly variable clinical trial results [8–10]. A recent meta-analysis indicated that PD-L1 expression is associated with improved ORR, OS, and PFS in ICI-treated mUC patients. However, the study underlined the limited NPV of the PD-L1 analysis, as about 20% of PD-L1-negative patients responded well to ICI therapy [31].

As currently, no real-world data are available on the predictive performance of PD-L1 IHC testing in real-life setting, the present work is the first to assess the ICI-predictive sensitivity, specificity, PPV, and NPV of the PD-L1 IHC test in mUC. Our findings showed a NPV of PD-L1 IHC of 55–70% (depending on the evaluation method), that is comparable to the results of the KEYNOTE-045/052 trials, while trials with atezolizumab (IMvigor210/211) displayed slightly higher NPVs (65–89%) [8, 32]. In the present study, the IC score outperformed the TC and CPS scores for ICI prediction in terms of specificity, sensitivity, PPV, and NPV of radiographic response. Furthermore, PD-L1 IHC correlated with its coding gene’s (*CD274*) expression but proved to be superior in its ICI-predictive performance.

Our GE analysis identified 23 genes with prognostic value for OS, and six of these could be validated in a large independent UC cohort (IMvigor210 study). According to ORR and DCR, 20 and 43 genes showed significantly different expressions, respectively. Although 7 genes (*C3, CXCL12, HLA-E, ICAM2, IRF7, MFGE8, PSMB10*) were associated with both endpoints (OS and DCR), *PSMB10* was the only one validated in the IMvigor210 transcriptome data for both outcomes.

The *PSMB10* (Proteasome Subunit Beta Type 10) gene encodes a component of the immunoproteasome (IP), a specialized proteasome involved in antigen processing. The IP is predominantly expressed in immune cells, including antigen-presenting cells and plays a critical role in the generation of peptide fragments presented by MHC class I molecules to cytotoxic T cells [33]. Recent studies in various cancer types have shown that overexpression of IP subunits (PSMB8, PSMB9, PSMB10) is associated with improved survival and better response to ICI therapies [34, 35]. Wang et al*.* demonstrated that the transcript levels of *PSMB8, PSMB9, PSMB10, PSME1, PSME2,* and *IRF1* are closely associated with CD8 + T lymphocyte levels in UC [36]. Additionally, they developed a predictive score (IP-score) based on these six genes to assess clinical response to ICI therapy. Notably, UC patients who responded to atezolizumab therapy exhibited a high IP-score compared to non-responders, and immune checkpoint genes, such as PD-1/PD-L1, were overexpressed in the high IP-score group [37]. Our results further validate the predictive value of IP-related genes (*PSMB8, PSMB10,* and *IRF1*). Future studies using functional bladder cancer models, molecular assays, activity-based probes, and single-cell approaches are needed elucidate PSMB10’s mechanistic role in tumor–immune interactions.

Gene expression-based molecular subtypes of UC have been suggested to show differences in treatment outcomes of ICI therapy. Kamoun et al*.* using the Consensus classification revealed, favorable responses to ICI (atezolizumab) in luminal nonspecified (LumNS), luminal unstable (LumU), and neuroendocrine-like (NE-like) tumors [34]. In the present study, applying various classification systems using a formerly published and independently validated gene panel-based approach, we found the highest ORR and longest OS in the neuronal subtypes, the TCGA–luminal-infiltrated, the Lund-mesenchymal, and the MDA p53-like subtypes; however, some of these associations did not reach the significance level because of the lower case number in less frequently occurring subtypes. Our observation that the TCGA–luminal-infiltrated subtype show better response to ICI therapy is consistent with the available literature showing that this subtype is characterized by high immune/stromal infiltration and high expression of PD-L1 thus is considered as ICI responsive subtype [35, 36]. However, further studies have shown that the TCGA luminal and the neuronal subtypes derive the most benefit from ICI therapy [37, 38]. Previous studies have suggested that tumors with a NE-like subtype might exhibit exceptionally favourable responses to ICI therapies [37, 38] (STable 12). Nevertheless, the generally low case numbers in this subgroup (representing > 10% of MIBC cohorts), possibly due to strict inclusion criteria, limit the robustness of these results. To address this issue, we examined neuronal signature score and found significant differences. High (≥ 3.2) neuronal scores were associated with improved OS and PFS in our ICI-treated UC cohort. This suggests that patients with tumors of a non-NE-like subtype but with elevated neuronal signature scores derive more benefit from ICI therapy.

Recently, integrative predictive models combining clinicopathological, laboratory, molecular, and genetic factors have gained interest for improving therapeutic decision-making.

Sonpavde et al. have proposed a risk stratification model incorporating ECOG PS, liver metastasis, platelet count, NLR, and LDH, which outperformed the Bellmunt model [38]. Similarly, in a study of patients with advanced UC treated with first-line ICIs, Khaki et al*.* included ECOG PS, albumin levels, NLR, and liver metastasis in their prognostic model [24]. A 4-tier model based on ECOG PS, alkaline phosphatase, hemoglobin, NLR, time from last chemotherapy, and liver or bone metastases also proved to show better performance in the risk stratification of UC patients compared to Bellmunt risk score. Notably, the inclusion of PD-L1 expression did not improve this model [27]. In addition to clinicopathological and laboratory markers, a 3-factor model by Nassar et al. include a genetic biomarker; TMB. According to this model, all patients who met the following three criteria—the absence of visceral metastases, NLR < 5, and high TMB—achieved a radiographic response (PR/CR) [39].

In an exploratory analysis, Bellmunt et al*.* investigated the relationship between PD-L1 expression, TMB, and two gene expression-based markers—T-cell–inflamed gene expression profile (TcellinfGEP) and the stromal signature—with pembrolizumab outcome in two KEYNOTE study populations. Their results showed that TMB and TcellinfGEP were significantly associated with improved outcomes in both cohorts [40]. Many other studies have assessed the predictive value of gene expression-based signatures [41], but there have been no reports on combining these factors with other biomarkers yet. In the present work, we developed a combination model that includes laboratory and gene expression-based factors that strongly enhanced the prognostic performance of standalone biomarkers. According to our results, patients with high-risk scores showed significantly worse OS compared to those in the low-risk score group (7.6 *vs.* 40.3 months, *p* = 0.002). These findings need to be further validated in independent cohorts, and in patients undergoing combination treatment.

We are aware that our research has some limitations. First, the retrospective nature of the study design limits the availability of the collected data. Second, our cohort was heterogeneous regarding the therapy line and applied ICI drug. Third, strict timing and central evaluation of CT/MRI scans were not feasible due to real-life circumstances. Therefore, treatment response was determined by the data collector based on clinical reports, which might influence the interpretation of the results. The modifying effect of chemotherapy on the composition of the tumor immune microenvironment could not be assessed, as most of the tumor samples used for gene expression analysis originated from primary lesions collected during radical surgery or TURB. PD-L1 IHC could not be included in the combined predictive models, as its data availability would significantly reduce the number of cases. A further limitation of our study is that the 14-parameter model could not be externally validated; while several single-gene associations were confirmed in the IMvigor210 dataset, the clinical parameters required for the integrated model were not available. As such, the predictive score should be regarded as preliminary until tested in independent, well-annotated cohorts. Finally, the predictive value of promising biomarkers, such as TMB or single-gene mutations, could not be assessed in our cohort because DNA sequencing was not performed.

On the other hand, this study has several key strengths. (1) This is one of the largest real-life studies of ICI-treated UC with respect to case number and number of the assessed genes. (2) The multicenter design of this study allowed for the inclusion of a diverse range of patients from real-world clinical practice. (3) This is the first study to investigate the sensitivity, specificity, and predictive values of PD-L1 IHC test in a real-life cohort of UC. (4) For molecular subtype classification, we successfully applied a simple gene panel-based method and used four different classification systems in the same cohort. (5) Through the combination of different parameters, we were able to increase the prognostic value of standalone markers.

In conclusion, the results of our real-life cohort analysis substantiate the limitations of the predictive value of standalone biomarkers, such as PD-L1, or molecular subtype classification. In addition, our study identified a series of gene expression-based markers (single genes and a gene expression signature) with a robust prognostic and/or predictive value for predicting outcomes of ICI treatment.

## Supplementary Information

Below is the link to the electronic supplementary material.

Supplementary file1

Supplementary file2

Supplementary file3

Supplementary file4

## Data Availability

All data supporting the findings of this study are available in the manuscript or supplementary materials. Additional data are available on reasonable request from the corresponding author.

## Abbreviations

1L: First-line
2L: Second-line
Ba/SCC-like: Basal/squamous cell carcinoma-like molecular subtype
Ba/Sq: Basal/squamous molecular subtype
BC: Bladder cancer
CI: Confidence interval
CR: Complete response (remission)
CRP: C-reactive protein
DCR: Disease control rate
DEG: Differentially expressed genes
DOR: Duration of response
ECOG PS: Eastern cooperative oncology group performance status
eGFR: Estimated glomerular filtration rate
FDR: False discovery rate
FFPE: Formalin-fixed, paraffin-embedded
GU: Genomically unstable molecular subtype
ICI: Immune checkpoint inhibitor
IHC: Immunohistochemistry
LDH: Lactate dehydrogenase
LN: Lymph node
Lum: Luminal molecular subtype
LumI: Luminal-infiltrated molecular subtype
LumNS: Luminal nonspecified molecular subtype
LumP: Luminal-papillary molecular subtype
LumU: Luminal unstable molecular subtype
MDA: MD Anderson Cancer Center
Mes-like: Mesenchymal-like molecular subtype
mUC: Metastatic urothelial carcinoma
NAC: Neoadjuvant chemotherapy
Ne: Neuronal molecular subtype
Ne-like: Neuroendocrine-like molecular subtype
NLR: Neutrophil–lymphocyte ratio
OS: Overall survival
ORR: Overall response rate
PD: Progressive disease
PD-1: Programmed death-1
PD-L1: Programmed death-ligand1
PFS: Progression-free survival
PR: Partial response
Sc/Ne-like: Small-cell/neuroendocrine-like molecular subtype
SD: Stable disease
TCGA: The cancer genome atlas
TMB: Tumor mutational burden
UC: Urothelial carcinoma
Uro-like: Urothelial-like molecular subtype
UTUC: Upper tract urothelial carcinoma
