# Supplementary material for: Combining molecular patterns and clinical data for better immune checkpoint inhibitor prediction in metastatic urothelial carcinoma
Source: Cancer Immunol Immunother. 2025 Nov 12;74(12):370. doi: 10.1007/s00262-025-04224-8 (PMC12612395; doi:10.1007/s00262-025-04224-8)
Supplement: Supplementary file 1 — Supplementary file1 [file 262_2025_4224_MOESM1_ESM.docx]

**Supplementary Material**

2. Material and methods

2.5. Statistical analysis

2.5.1 Single marker analysis

Descriptive statistics included median and range for continuous variables and numbers and percentages for categorical variables. Cox proportional hazard models were used to assess differences in hazard ratios (HR) between groups according to the risk factors and stratified two-sided log-rank test with Kaplan-Meier plots were used to evaluate OS and progression-free survival (PFS). OS was defined as the period from the initiation of ICI therapy to the date of death from any cause, while PFS was defined as the time from the first ICI treatment to the date of disease progression (radiographic or clinical) or death from any cause. Chi-square test was used to determine the association between objective response (CR/PR) or disease control (CR/PR/SD) rates and different clinicopathological variables. Correlation between PD-L1 IHC scores and CD274 gene expression was assessed using Pearson’s correlation. For all methods, two-sided p-values ≤0.050 were considered statistically significant. These statistical analyses were performed with SPSS version 27.0 (IBM, Armonk, N.Y., USA)

NanoString data was analyzed within the R for Windows environment (v4.4.0, R Foundation for Statistical Computing, Vienna, Austria, 2024). In addition to the manual calculation of log2 fold change (log2FC), the comparison of normalized NanoString data (differential expression analysis) was completed using Wilcoxon rank sum tests. If a parameter had more than two levels to compare, pairwise Wilcoxon rank sum tests with Holm corrected p-values (1) were used. For the data obtained from the nCounter® PanCancer Immune Profiling Panel, gene set enrichment analysis was also feasible to perform. Survival analysis was performed using Cox regression models. Optimal cut-off values for each of the genes were calculated according to the following process: first, the upper (Q3) and lower quartiles (Q1) of expression values of each gene were determined, then the best cut-off value between the upper and lower quartiles was identified for each gene by evaluating all possible cut-off values in the Cox regression analysis and finally computing the Benjamini-Hochberg False-Discovery Rate to correct for multiple hypothesis testing (2). The Kaplan-Meier plots were drawn by using the best cut-off. The results were drawn using boxplots and heatmaps with the ggplot2 (version 3.5.1) and the ComplexHeatmap (version 2.20.0) R-packages, respectively.

For the validation of the prognostic value of selected genes, the online tool "Kaplan-Meier Plotter" (www.kmplot.com; accessed on 09/06/2024) was used. This tool analyses publicly available gene expression and survival data from multiple cancer datasets, including those with ICI-treated UC patients (3). In addition, the "ROC Plotter" (www.rocplot.com/immune; accessed on 09/06/2024), was used to assess the diagnostic and predictive performance of differentially expressed genes (DEGs). The ROC Plotter database includes 1,434 tumor tissue samples from 19 datasets covering esophageal, gastric, head and neck, lung, melanoma, and UC cancers (4). Both pan-cancer and UC-specific (IMvigor210) validations were performed.

2.5.2 Combination Model Development

Data Analysis

All analyses were performed using Python (version 3.12.7) programming language and environment. Feature selection, model training, and evaluation were conducted using the scikit-learn library (version 1.5.1). Survival analyses were carried out with the lifelines package (version 0.29.0), while specialized survival models were implemented using scikit-survival (version 0.23.0).

Data preprocessing

The data preprocessing pipeline involved handling missing values and preparing features for analysis. Missing values in numeric columns were imputed using the median, while categorical columns were imputed using the mode to ensure robustness and minimize bias. The dataset included PD-L1 IHC results, NanoString gene expression data, signature scores, and clinical variables such as sex, age at ICI initiation, Eastern Cooperative Oncology Group performance status (ECOG PS), Bellmunt risk scores, and metastasis types (liver, visceral, LN-only, and bone). Additional clinical and laboratory parameters considered were CRP, LDH, NLR, Hg, albumin, and eGFR.

Survival analysis

For survival analysis, features were first filtered based on univariable survival metrics to retain only clinicopathological and molecular factors that were significantly associated with OS in univariate analysis; additionally, each parameter was required to be available for at least 80% of patients to ensure adequate sample size. The dataset was split into training (75%) and testing (25%) subsets. Random Survival Forest (RSF) model was employed with 50 trees (hyperparameters: minimum of samples per split: 8, minimum of samples per leaf: 4). The RSF model was trained on the training data, and its performance was evaluated on the test set using the concordance index (C-index) as the metric for survival prediction accuracy (SFigure 4). We further refined the predictors by evaluating their contribution within a Random Survival Forest framework. Predictors with consistently low usage were considered less informative and were iteratively removed, retraining the model after each reduction step.

The predicted survival scores were used to stratify the population into low- and high-score groups based on the median score. A Cox Proportional-Hazards model was fitted to assess the association between predicted survival scores and observed survival outcomes.


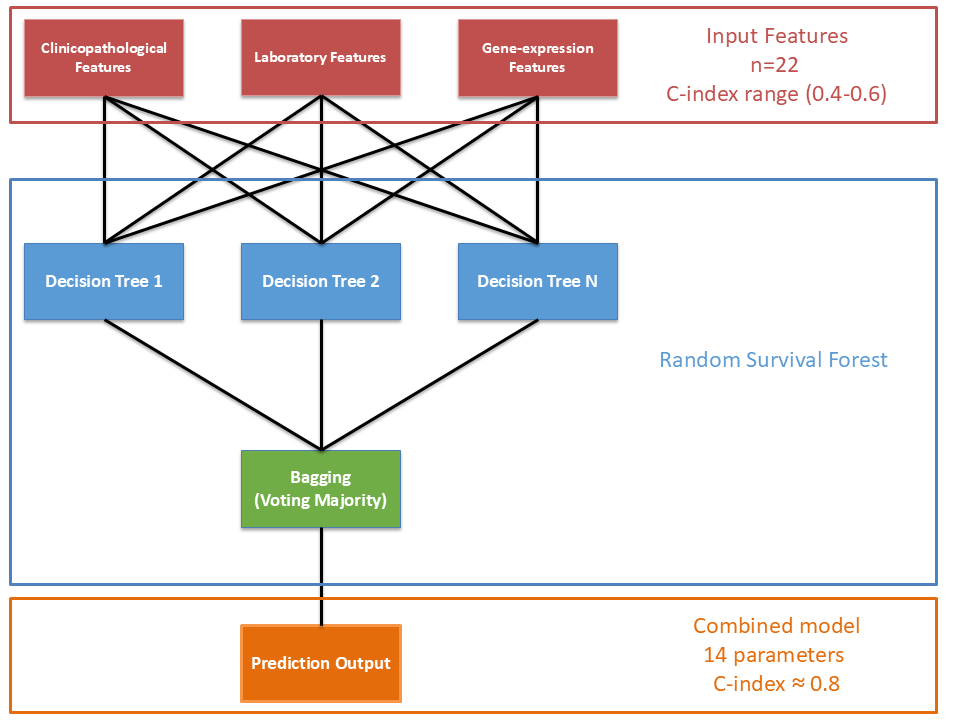


SFigure 4: Workflow of Random Survival Forest Model

References:

1. Holm S. A Simple Sequentially Rejective Multiple Test Procedure. Scandinavian Journal of Statistics. 1979;6(2):65-70.

2. Benjamini Y, Hochberg Y. Controlling the False Discovery Rate: A Practical and Powerful Approach to Multiple Testing. Journal of the Royal Statistical Society. Series B (Methodological). 1995;57(1):289-300.

3. Gyorffy B. Integrated analysis of public datasets for the discovery and validation of survival-associated genes in solid tumors. Innovation (Camb). 2024;5(3):100625. <https://doi.org/10.1016/j.xinn.2024.100625>.

4. Kovacs SA, Fekete JT, Gyorffy B. Predictive biomarkers of immunotherapy response with pharmacological applications in solid tumors. Acta Pharmacol Sin. 2023;44(9):1879-89. <https://doi.org/10.1038/s41401-023-01079-6>.
