# Supplementary figures and images for: Combining molecular patterns and clinical data for better immune checkpoint inhibitor prediction in metastatic urothelial carcinoma
Source: Cancer Immunol Immunother. 2025 Nov 12;74(12):370. doi: 10.1007/s00262-025-04224-8 (PMC12612395; doi:10.1007/s00262-025-04224-8)

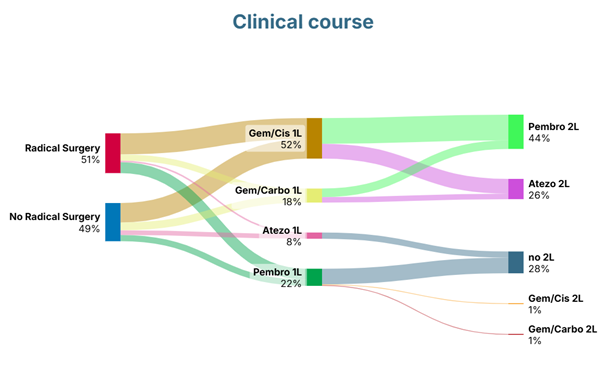

Supplement: Supplementary file 3 — Supplementary file3 [file 262_2025_4224_MOESM3_ESM.png]

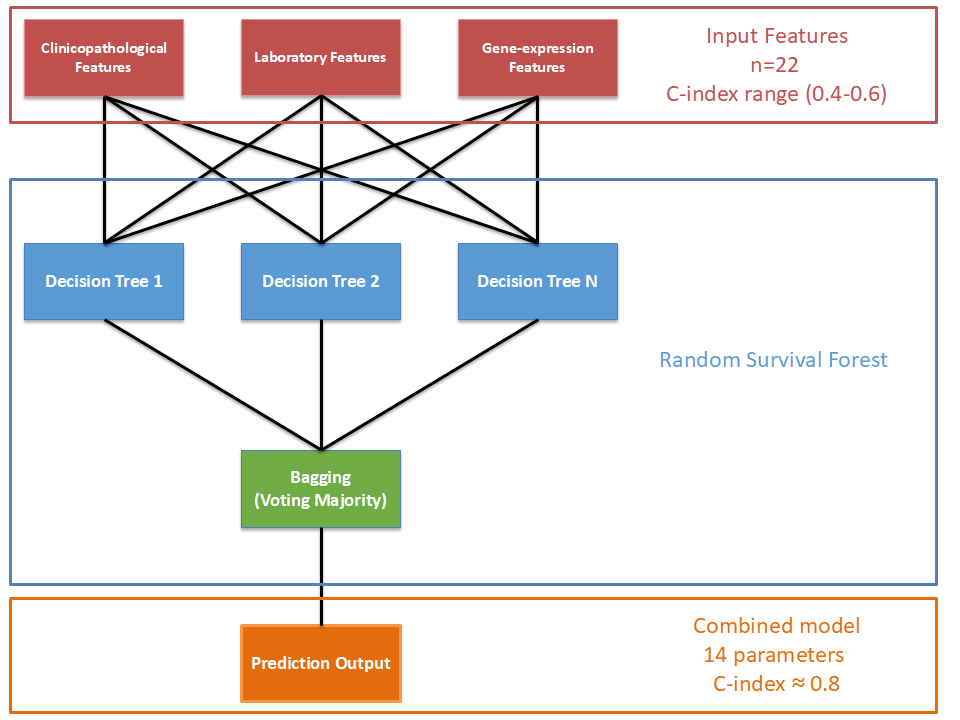

Supplement: Supplementary file 4 — Supplementary file4 [file 262_2025_4224_MOESM4_ESM.tif]
